# Microtensile bond strength of bulk-fill restorative composites to dentin

**DOI:** 10.4317/jced.53965

**Published:** 2017-08-01

**Authors:** Jyothi Mandava, Divya-Prasanna Vegesna, Ravichandra Ravi, Mohan-Rao Boddeda, Lakshman-Varma Uppalapati, MD Ghazanfaruddin

**Affiliations:** 1 Professor and Head of the Department, Department of Conservative dentistry, GITAM Dental college and hospital, Visakhapatnam; 2 Post Graduate student, Department of Conservative dentistry, GITAM Dental college and hospital, Visakhapatnam; 3 Senior lecturer, Department of Conservative dentistry, GITAM Dental college and hospital, Visakhapatnam; 4 Reader, Department of Conservative dentistry, GITAM Dental college and hospital, Visakhapatnam

## Abstract

**Background:**

To facilitate the easier placement of direct resin composite in deeper cavities, bulk fill composites have been introduced. The Mechanical stability of fillings in stress bearing areas restored with bulk-fill resin composites is still open to question, since long term clinical studies are not available so far. Thus, the objective of the study was to evaluate and compare the microtensile bond strength of three bulk-fill restorative composites with a nanohybrid composite.

**Material and Methods:**

Class I cavities were prepared on sixty extracted mandibular molars. Teeth were divided into 4 groups (n= 15 each) and in group I, the prepared cavities were restored with nanohybrid (Filtek Z250 XT) restorative composite in an incremental manner. In group II, III and IV, the bulk-fill composites (Filtek, Tetric EvoCeram, X-tra fil bulk-fill restoratives) were placed as a 4 mm single increment and light cured. The restored teeth were subjected to thermocycling and bond strength testing was done using instron testing machine. The mode of failure was assessed by scanning electron microscope (SEM). The bond strength values obtained in megapascals (MPa) were subjected to statistical analysis, using SPSS/PC version 20 software.One-way ANOVA was used for groupwise comparison of the bond strength. Tukey’s Post Hoc test was used for pairwise comparisons among the groups.

**Results:**

The highest mean bond strength was achieved with Filtek bulk-fill restorative showing statistically significant difference with Tetric EvoCeram bulk-fill (*p*< 0.003) and X-tra fil bulk-fill (*p*<0.001) composites. Adhesive failures are mostly observed with X-tra fil bulk fill composites, whereas mixed failures are more common with other bulk fill composites.

**Conclusions:**

Bulk-fill composites exhibited adequate bond strength to dentin and can be considered as restorative material of choice in posterior stress bearing areas.

** Key words:**Bond strength, Bulk-fill restoratives, Configuration factor, Polymerization shrinkage.

## Introduction

With rapid improvements in material science, there is now wide spread use of composite resins for restoration of posterior teeth even in stress bearing areas (1). All direct esthetic restorations are bonded to tooth structure and generating an effective bond is paramount for the success and longevity of such restorations (2). Many factors like amount of residual tooth structure available for bonding, use of appropriate composite placement, curing techniques and occlusal force equilibration contribute to achieve clinical success of direct posterior composite restoirations (3). However, an annual failure rate of posterior composite restorations range from 0%-9% (4).

Composite polymerization produces internal stresses which could lead to bond loss at the tooth composite interface, cuspal deflection, and enamel crack formation, all of which are primary factors in the potential failure of a restoration (5). Limitation in depth of cure causes incomplete polymerization with possibility of insufficient monomer conversion resulting in inferior physical and biological properties of resin composites (6). Therefore, several attempts have been made to minimise the amount of polymerization stress generated and to maximize the depth of cure through changing the formulations.

Bulk-fill composites have been introduced to overcome some of the shortcomings of light cure resin composites. These materials are suitable for insertion in a 4mm bulk placement due to their reduced polymerization stress and their high reactivity to light curing. The higher depth of cure of these materials is due to the presence of different photo-initiators that are more translucent and allow the light to pass through much deeper layers (7). The low polymerization stresses for the bulk-fill composites are due to modification in the filler content and / or organic matrix and also due to the presence of stress inhibitors (8). They have shown reduced cuspal deflection when compared with a conventional resin composites filled in an oblique incremental layering technique (9) and also when marginal integrity was evaluated, bulk-fill composites performed well (10). The longevity of these newly introduced restorative materials and their bond strength to the tooth structure is less studied.

An effective bonding to tooth structure eliminates the microleakage by sealing the dentinal tubules and restorative margins, and thus preventing the adverse consequences of post restorative hypersensitivity, marginal discolouration, recurrent caries and harmful effects on the pulp (11). Usually laboratory bond strength testing is done to demonstrate the quality of dentin adhesion of the newer materials relative to their competitors (12). The rationale behind this testing method is that the stronger the adhesion between tooth and biomaterial, better will the resistance offered by a restoration to stresses imposed by resin polymerization and oral function. The microtensile bond strength testing (µTBS) has several advantages over conventional bond strength testing methods, as this method gives an opportunity to investigate interfacial bond strengths on small areas of below 1mm2 (13,14). This renders this test more versatile, as multiple specimens can be obtained from a single tooth enabling more inventive study setups and better controlled substrate variables (5).

Thus, the present study was conducted to evaluate and compare the micro-tensile bond strength of three bulk-fill restorative composites with a nanohybrid composite and to assess the mode of failure at the resin dentin interface. The null hypothesis was there will be no difference in the microtensile bond strength or in failure modes of evaluated bulk-fill composites.

## Material and Methods

Sixty, extracted human mandibular molar teeth with approximately similar mesiodistal and buccolingual dimensions without any caries, cracks, restorations or structural deformities were used in the study. Collected teeth were freshly extracted due to periodontal problems and their use in research was approved by the local biomedical research ethics committee (D148601060). The teeth were stored in 0.5% chloramine-T, that was replaced once in 15 days to avoid contamination.

On each molar, occlusal class I cavities were prepared using a high speed handpiece and diamond burs (EX-41, Dia burs-MANI, Tochigi, Japan) with air and water coolant. The final dimensions of the prepared cavity were approximately 3.5 mm wide, 4 mm deep with a configuration factor (C-factor) of 5.0. Tooth samples were randomly divided into four groups (n=15), according to the type of composite used for restoring class I cavities ( Table 1). Etching and bonding procedures were done according to the manufacturer’s instructions. In group I, horizontal incremental layering technique was used to fill the cavity with Filtek Z250 XT (3M ESPE, St. Paul, MN, USA) nanohybrid composite and light activation was done with LED light curing unit (Bluephase C8, Ivoclar Vivadent, Liechtenstein, USA) with an intensity of 800 mW/cm2, for 20 seconds.

**Table 1:**
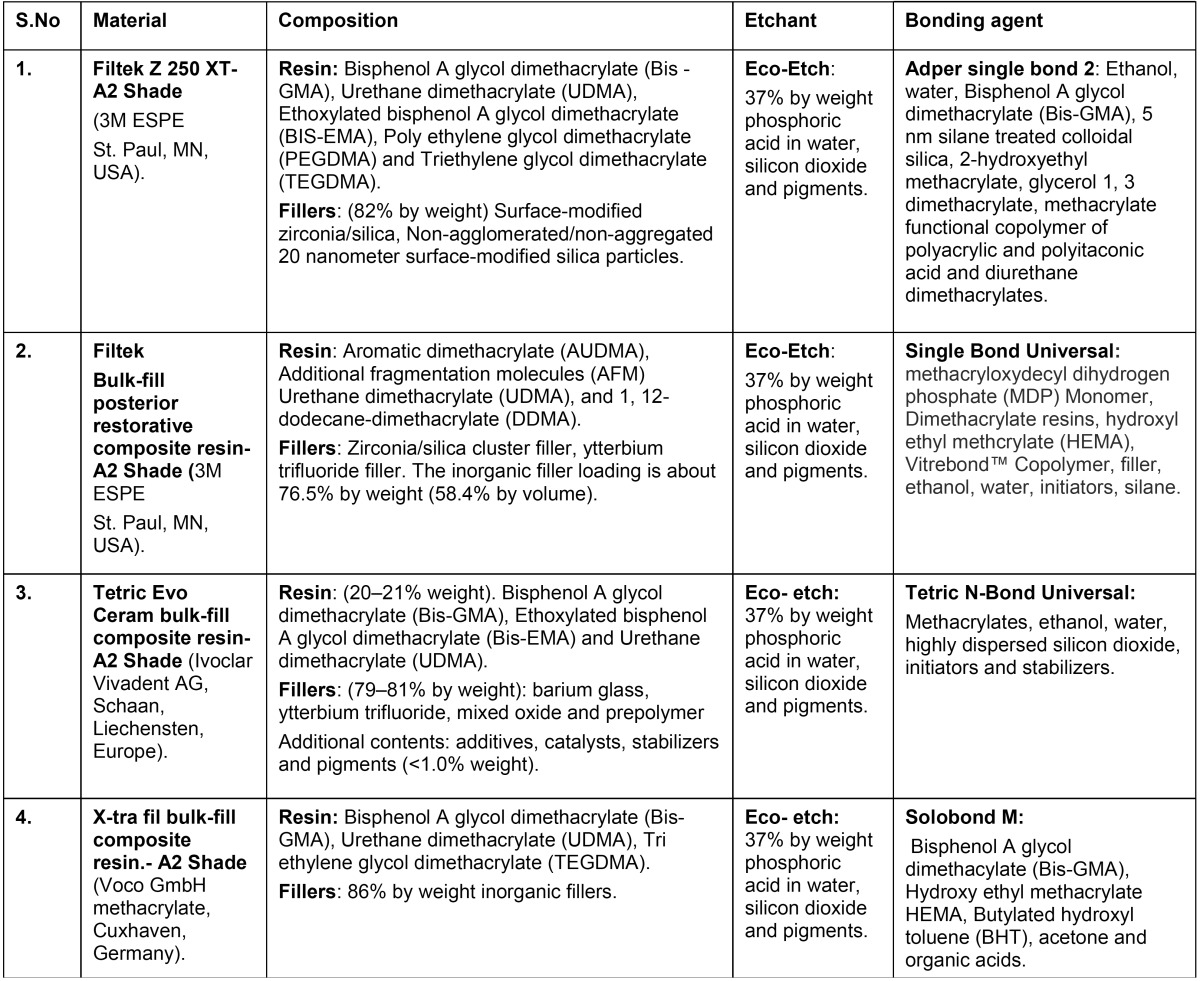
Composition of the materials used in the study.

The prepared cavities in group II were restored with Filtek bulk-fill posterior restorative (3M ESPE, St. Paul, MN, USA); group III, with Tetric EvoCeram bulk-fill (Ivoclar Vivadent, Liechensten, Europe) and in group IV with X-tra fil bulk-fill (Voco, Cuxhaven, Germany) composite resins. In these three groups, the composite was placed as a single increment of 4mm thickness into the prepared cavities and light cured for 20 seconds. The restorations were then finished with diamond finishing burs (TR-25EF, Mani, Japan) under abundant air water spray and were polished using Sof-lex XT polishing and finishing system (3M ESPE, St. Paul, MN, USA).

**Figure 1:**
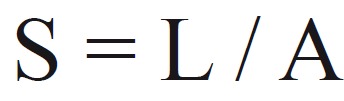
Formula.

The restored teeth were stored in an incubator with 100% humidity at 370C for 1 week and were subjected to thermocycling (Wileytec thermocycler, Haake ek 30, Germany) for 10,000 cycles in water bath between 50 and 550 with a dwell time of 30 seconds and a transfer time of 5 seconds. The teeth were then mounted in acrylic resin blocks and a low speed diamond saw (Hard tissue microtome, Leica SP 1600, Germany) was used to section the teeth under copious water coolant. Two cuts were made in a mesio-distal direction along the long axis of the teeth with a 1 mm thick diamond disc and then the center restorative part of the tooth was sectioned bucco-lingually by giving four cuts. Thus, three bonded stick shaped specimens of 0.9 mm ± 0.1 mm2 cross -sectional area were obtained from each tooth. A total of 180 specimens (n= 45 each group) obtained from all the groups were subjected to bond strength evaluation using universal testing machine (Autograph, AG- 15, Shimadzu inc, USA). Each beam was attached to a custom made jig using cyanoacrylate glue and a tensile load was applied at a cross head speed of 1 mm/min until the beam fractured. The amount of load required for fracture recorded in newtons was converted to megapascals (Mpa) by using the formula, (Fig. 1).

Where S is the bond strength in mega pascals (MPa)

L= test load (N)

A= adhesive area (mm2) 

To assess the mode of failure the fractured specimens were examined under scanning electron microscope (JOEL JSM 5600, MA, USA). Photomicrographs were taken at 200X magnification and the failure mode (cohesive, adhesive or mixed failure) was identified for each specimen.

Statistical analysis

The bond strength values obtained were subjected to statistical analysis using SPSS/PC version 20 software. A one-way analysis of variance (ANOVA) was used to compare the forces at which fracture occurred. Tukey’s Honest significant difference Post Hoc test was used for comparisons among the four groups. The statistical analysis was performed at 95% confidence intervel.

## Results

**Figure 2:**
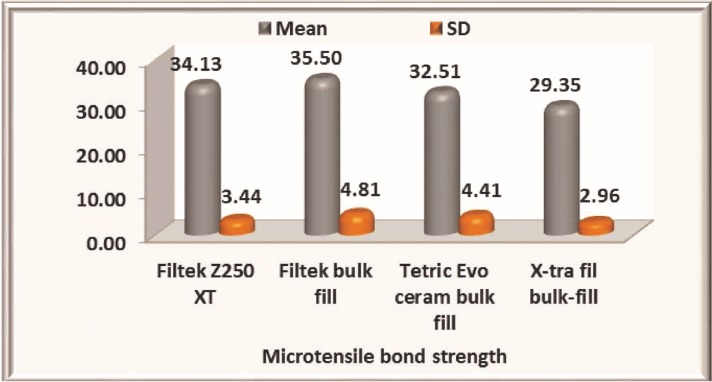
Bar diagrammatic representation of mean bond strength and standard deviation in Mpa.

The mean bond strength values obtained for different groups (Fig. 2). are decreased in the following order; Filtek bulk-fill > Filtek Z 250 XT > Tetric EvoCeram bulk-fill > X-tra fill bulk-fill. The microtensile bond strength of group IV (X-tra fil bulk-fill) is significantly low (*p*=0.001) compared to all other three groups ( Table 2). Filtek bulk-fill has shown highest mean bond strength showing no statistically significant difference when compared to nanohybrid (*p*=0.367) composite. Significant difference in bond trength was observed between Filtek bulk fill and Tetric Evoceram bulk fill (*p*=0.003) composites.

**Table 2:**
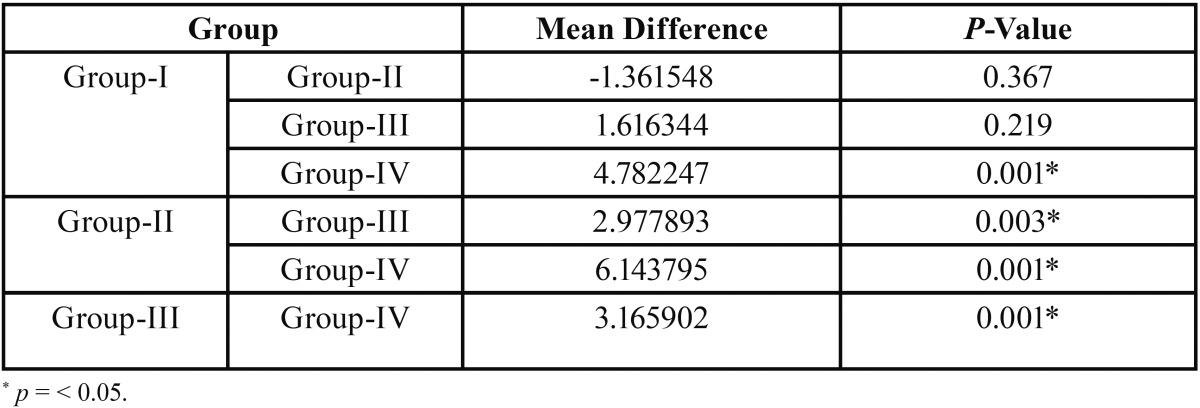
Tukey’s HSD Post Hoc Test for multiple comparisons between groups.

**Figure 3:**
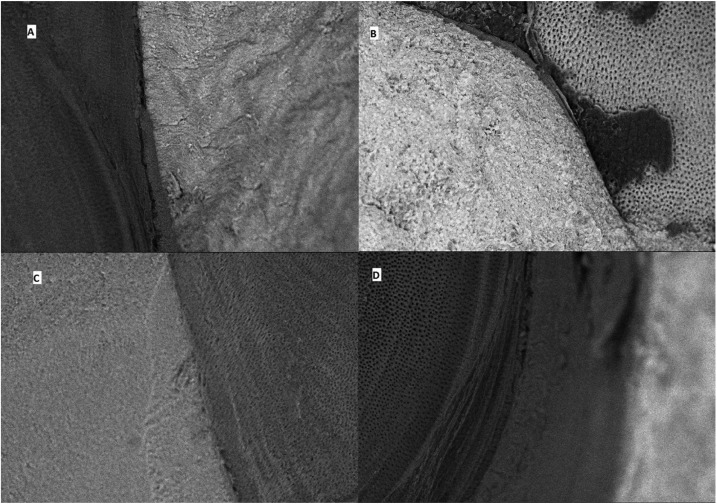
Scanning electron microscopic images of group I showing mixed failure (A), group II showing mixed failure (B), group III: mixed failure, and group IV showing adhesive failure.

SEM examination revealed more number of mixed failures in Filtek bulk-fill, Tetric EvoCeram bulk-fill and nanohybrid composites (Fig. 3). Maximum number of samples restored with X-tra fil bulk-fill exhibited adhesive failures, correlating with the lowest values obtained in bond strength testing.

## Discussion

Microtensile bond strength of newly introduced bulk-fill composites were evaluated as formation of the adhesive bond to tooth structure is the most important factor for the long term retention of composite restorations (15). Three commercially available bulk-fill composites were compared with a nanohybrid universal restorative composite having high filler loading (82% wt% and 68% vol%), with improved mechanical properties and better clinical performance (16,17).

Class I cavities with high configuration factor were prepared as the resultant stress put the resin-tooth interface under increased tension as there is less chance for relaxation of shrinkage stresses. It has been reported in several studies that the increase in C-factor is associated with a progressive decrease in bond strength leading to a potential deleterious effect on marginal integrity and gap formation (18,19). As in vitro evaluation of restorative materials fails to simulate the intra-oral thermal changes during eating and drinking, thermocycling was performed for 10,000 cycles, that corresponds approximately to 1 year of *in vivo* functioning (14).

The proposed null hypothesis was rejected as there were significant differences in the bond strength values exhibited by the tested bulk fill restorative resins. The newly introduced Filtek bulk-fill posterior restorative have shown highest mean microtensile bond strength among all the test groups, which can be attributed to the presence of modified resin matrix that lowers the shrinkage stress as proposed by the manufacturer. The resin matrix of Filtek bulk-fill posterior consists of aromatic dimethacrylate (AUD-MA), additional fragmentation molecules (AFM), urethane dimethacrylate (UDMA) and 1,12-dodecane dimethacrylate (DDMA). The inclusion of these monomers into the polymerization mixture enables the network to rearrange, and get adapted during and/or after the polymerization, to accommodate the shrinkage without developing significant stresses (19,20). Apart from this, Filtek Bulk-Fill contains additional zirconia filler and substitution of glass fillers with zirconia/ silica fillers (2.5 and 5.0 wt%) is said to improve mechanical properties, such as flexural strength and fracture toughness (21).

Lower bond strength obtained for Tetric EvoCeram bulk-fill restoratives compared to nanohybrid group (Filtek Z250 XT) might be due to lower filler loading of the former. The nanohybrid resin composite had mean flexural modulus (17.44 Gpa) similar to the flexural modulus of dentin (19 GPa) that can be effective in longevity of restorations (22). It was reported that if filler content was increased with decreasing particle size and interparticle spacing, the fatigue limit of the material improves due to increased obstacles for crack growth (23). In addition, the nanocluster particles of Filtek Z 250 XT possess different mechanical properties compared with filler particles seen in the spherical mixed oxide and isofillers of Tetric EvoCeram bulk-fill. The shape of Tetric EvoCeram bulk-fill fillers approaching round-shape were shown to positively influence the translucency to improve the depth of cure (24) but compromised the mechanical properties when compared to the nanohybrid composites.

X-tra fil bulk-fill exhibited the lowest mean bond strength when compared to the other groups. Correlating with our results, a study by Damanhoury H *et al.* (25) revealed that the shrinkage stress generated by X-tra fil bulk-fill was significantly higher than the Tetric EvoCeram bulk-fill which could be related to the lower bond strength achieved with X-tra fil bulk-fill composite. The magnitude of polymerization shrinkage stress has been found to be dependent on volumetric polymerization shrinkage and polymer elastic modulus. Whereas, polymerization shrinkage is related to the degree of conversion and initial reactive group concentration (26). Generally, increasing the filler load in the resin matrix results in reduction of overall shrinkage of composite due to reduced availability of the monomer for the curing reaction. But it also may result in a high elastic modulus of the material, which can lead to high shrinkage stress (9). In a study by Behery H *et al.* (27) lower mean cuspal deflection value was seen for Tetric EvoCeram bulk-fill when compared to X-tra fil bulk-fill composite and that was attributed to low elastic modulus of Tetric bulk fill (10 GPa) in comparison to X-tra fil (16 GPa) as assessed by Damanhoury H *et al.* (25).

SEM observation allows for analyzing whether the methodology used provides bond strength values that correspond to the adhesive-dentin bond interface or not. The least microtensile bond strength values were seen with X-tra fil bulk -fill composite which can be justified with the predominant adhesive failures observed with SEM examination in this group. The least number of cohesive failures and more of mixed failures exhibited by other bulk-fill resin composites might be attributed to the improved depth of cure.

A key factor for a clinically effective and durable composite resin restoration is to maintain stable bond and leak proof tooth-restorative margins (13,14). *In-vitro* testing shows different degrees of clinical relevance (13,28) and more clinically relevant bond strength testing necessitates the stimulation of oral environmental factors like masticatory forces and pH fluctuations of the saliva.

## Conclusions

All the bulk-fill restoratives tested in the study exhibited the mean bond strength values around 30 Mpa. This may support the intended use of these materials for bulk filling the deep class I cavities with high C factor.
